# Physical activity, sex differences, and functional exercise capacity in bronchiectasis: Insights from a South Asian cohort

**DOI:** 10.1371/journal.pone.0356560

**Published:** 2026-09-02

**Authors:** Anup Bhat, Annemarie L. Lee, G. Arun Maiya, Mohan K. Manu, Aswini Kumar Mohapatra, Rahul Magazine, K. Vaishali

**Affiliations:** 1 Department of Physiotherapy, Manipal College of Health Professions, Manipal Academy of Higher Education, Manipal, India; 2 Department of Physiotherapy, School of Primary and Allied Health Care, Monash University, Frankston, Victoria, Australia; 3 Institute for Breathing and Sleep, Heidelberg, Victoria, Australia; 4 Department of Respiratory Medicine, Kasturba Medical College, Manipal Academy of Higher Education, Manipal, India; Kurume University School of Medicine: Kurume Daigaku Igakubu Daigakuin Igaku Kenkyuka, JAPAN

## Abstract

Bronchiectasis causes breathlessness and fatigue, reducing quality of life. Many affected individuals are less active than healthy peers, increasing their risk of exacerbations and hospitalizations. Correlates of physical activity (PA) and functional exercise capacity remain inconsistent, and most evidence comes from Western populations. Sex-specific differences in disease presentation and functional outcomes are recognised yet remain underexplored in South Asian cohorts. The aim of the study was to examine the relationship between PA and demographic, clinical, and functional variables; to compare these characteristics between sexes; and to identify predictors of functional exercise capacity in individuals with bronchiectasis. Fifty-five adults with stable bronchiectasis were assessed using the International Physical Activity Questionnaire for PA, the modified Medical Research Council scale (mMRC) for dyspnoea, the six-minute walk distance (6MWD) for functional exercise capacity and the bronchiectasis health questionnaire (BHQ) for health status. Bronchiectasis-related clinical characteristics, including disease severity, exacerbation frequency, microbiological status, radiological severity, and disease duration were also recorded. Associations and predictors were examined using correlation analyses and multivariate regression. Lower PA was associated with greater dyspnoea severity (rs = −0.344) and shorter disease duration (rs = 0.275). Females had shorter absolute walking distance (≈57 metres shorter 6MWD) but no significant difference in % predicted 6MWD compared with males. Females also reported higher PA (+1350 MET-minutes/week) than males. Reduced 6MWD correlated with older age, lower BHQ score, female sex, increased exacerbation frequency, greater dyspnoea severity and higher radiological severity. Dyspnoea severity and radiological severity independently predicted functional exercise capacity. Lower PA was weakly associated with greater dyspnoea severity and shorter disease duration. Females reported higher self-reported PA levels despite shorter absolute walking distances than males. Dyspnoea severity emerged as the strongest predictor of functional exercise capacity, while radiological severity was also associated with reduced 6MWD.

## Introduction

Bronchiectasis is a chronic respiratory syndrome that causes bronchial dilatation leading to a complex cycle of impaired mucociliary clearance, inflammation, infection, and further structural damage to airways [1,2]. Bronchiectasis presents with cough, sputum production, and breathlessness, which impair exercise capacity and quality of life [3]. Physical inactivity is common and may result from respiratory symptoms, fatigue, and social embarrassment [3–6].

Individuals with bronchiectasis are less active than healthy peers, and this inactivity has been linked to increased exacerbations and hospitalizations [5–9]. A nuanced understanding of factors contributing to reduced physical activity (PA) in adults with bronchiectasis is clinically relevant because improving exercise capacity alone does not necessarily increase PA [10]. In bronchiectasis, studies using wearable PA monitors have generally shown that lower PA is associated with greater dyspnoea [6,11,12], and poorer functional exercise capacity [5–7,11–13]. Some have also reported links with health status [7,14], although findings are inconsistent. Questionnaire-based PA assessments [15] are underused, even though simple validated tools offer a practical option when device-based monitoring is difficult to implement in many South Asian health care contexts. These tools reduce patient burden and are feasible for both research and routine evaluation. Wearable PA monitors are often impractical because of travel demands, out-of-pocket costs and limited follow-up opportunities. Evidence on the factors influencing PA in bronchiectasis within these contexts remains scarce. Understanding whether demographic, clinical or functional variables are associated with lower levels of activity in patients could inform clinicians of targeted interventions to address this limitation.

Beyond general factors influencing PA and assessment methods, it is also crucial to consider demographic specificities. Emerging evidence [16–18] in adults with bronchiectasis indicates that disease manifestation and functional exercise capacity differ by sex, but few have examined these patterns in South Asian populations. Sociocultural and occupational roles in this region may influence activity patterns and symptom perception differently than in Western cohorts. Characterizing such differences could help design more tailored pulmonary rehabilitation programs.

Functional exercise capacity is a key outcome in bronchiectasis [3,5,11], as it reflects patients’ ability to perform daily activities. Prior studies have identified potential determinants of functional exercise capacity including demographic factors [16,19] clinical measures [17–21], PA levels [21] and health status or quality of life measures [18,21], but their findings remain inconsistent. Some reports suggest that older age predicts lower capacity [16], whereas others associate lower capacity with younger age [19] or find no relationship at all [20]. Similarly, quality of life has been linked to exercise capacity in some studies [18,21], while others have shown no association [16,17,19]. Most available evidence comes from Western cohorts, whereas clinical presentation and disease behaviour in Asian populations differ in bronchiectasis [22], underscoring the need for region-specific data to identify factors influencing functional exercise capacity in bronchiectasis.

The present study therefore aimed to (1) examine the relationships between PA and demographic features, clinical characteristics and functional characteristics in individuals with stable bronchiectasis; (2) compare these characteristics between sexes; and (3) identify predictors of functional exercise capacity. These insights, derived using clinically scalable tools, may guide the design of individualized pulmonary rehabilitation strategies.

## Materials and methods

This cross-sectional analysis used baseline data from a randomized controlled trial titled “Effectiveness of structured home-based pulmonary rehabilitation program on functional capacity and quality of life in bronchiectasis: a randomized controlled trial” (CTRI/2020/01/022893), conducted at the Manipal Academy of Higher Education, India. The study was approved by the Institutional Ethics Committee (IEC: 135/2019) for the period from 12 February 2019 to 11 February 2023. Participant recruitment and data collection were initiated during this period, beginning on 23 July 2021. Following a temporary pause, an extension of ethical approval was obtained from 6 August 2023 to 15 January 2025, after which data collection was resumed and completed on 31 July 2024. All data were collected during periods covered by valid ethical approval. Written informed consent was obtained from all participants prior to recruitment.

Participants were recruited from a single tertiary care hospital as part of the baseline assessment of the trial. Most participants were recruited from outpatient respiratory medicine services, with a small number recruited during hospital admission for diagnostic evaluation. Approximately 220 individuals with bronchiectasis were followed at the service during the study period. A total of 187 individuals were screened for eligibility, of whom 55 participants meeting the study criteria were included in the present analysis.

Stable individuals with bronchiectasis diagnosed clinically by treating pulmonologists and a modified Medical Research Council (mMRC) dyspnoea grade greater than 1 were included in the study. Most participants (n = 53) had radiological confirmation of bronchiectasis using high-resolution computed tomography (HRCT), with scans interpreted by qualified radiologists in accordance with standard radiological criteria. In one participant, the HRCT report was unavailable despite a prior radiological diagnosis. In another participant, HRCT had not been performed, and the diagnosis was based on the treating pulmonologist’s clinical assessment, chest radiographic findings, and available medical records. Individuals with neuromuscular, musculoskeletal, or cardiovascular impairments limiting exercise participation were excluded.

### Data collection and measurements

Demographic and clinical details including age, sex, occupation, height, weight, body mass index (BMI), disease severity, duration since diagnosis, exacerbation frequency, microbiological status, radiological severity, cause of bronchiectasis, comorbidities, and medications were collected from medical records and participant interviews during the initial baseline evaluation. Exacerbation frequency was recorded as the number of bronchiectasis exacerbations in the preceding year. Microbiological status was determined from available sputum culture reports. Radiological severity was classified using the radiological component of the bronchiectasis severity index [23] based on the number of involved lobes and the presence of cystic bronchiectasis. Etiological evaluation was based on clinical history, radiological findings, and available medical records. Microbiological assessment included routine respiratory bacteriology and frequent evaluation for mycobacterial infection. Allergic bronchopulmonary aspergillosis (ABPA) was investigated in patients with clinical features suggestive of asthma. HIV screening was performed as part of routine evaluation. Investigations for less common etiologies, including cystic fibrosis and primary ciliary dyskinesia, were not routinely performed but were considered in patients with suggestive clinical features. Immunoglobulin levels were not systematically assessed in all participants.

Disease severity was categorised using the bronchiectasis severity index (BSI). In participants with incomplete data required for conventional BSI calculation, a relative BSI categorisation approach described by Howarth et al.[24] was applied. This modified approach was used when one or more BSI components, including forced expiratory volume in 1 second as a percentage of the predicted value, sputum microbiology or high-resolution computed tomography were unavailable. In such instances, a relative BSI score was calculated using the available variables, and participants were categorised into mild, moderate, or severe disease categories.[24] For analysis, only the disease severity categories were used, not the raw BSI scores. This approach was applied to our dataset because similar pragmatic challenges related to the availability of diagnostic investigations have previously been described in the adult Aboriginal Australian cohort reported by Howarth et al., [24]. Incomplete BSI data were present in 21 participants. Missing components included FEV1 alone (n = 14), microbiology alone (n = 3), HRCT alone (n = 1), microbiology and FEV1 (n = 2), microbiology and HRCT (n = 1). In our setting, some participants travelled long distances for specialist evaluation. Additional investigations were occasionally deferred because of out-of-pocket healthcare expenditure, inability to perform spirometry, or participant preference against undergoing further testing.

Health status was assessed using the BHQ, a validated brief 10-item disease specific health status questionnaire that provides a single score from 0–100 [25]. A score of 100 indicates the best health status [25].

Functional exercise capacity was evaluated using the distance covered in the six-minute walk test (6MWD) performed on a 30-metre corridor in accordance with American Thoracic Society guidelines [26,27]. Heart rate, respiratory rate, blood pressure, oxygen saturation and the rate of perceived exertion according to the Borg rating of the perceived exertion scale [28] were recorded at baseline, immediately after the test, and monitoring continued until readings returned to baseline. The 6MWD was additionally expressed as a percentage of predicted values (% predicted 6MWD), calculated using reference equations derived from an Indian population [29], incorporating age and sex.

Physical activity was assessed using the international physical activity questionnaire – short form (IPAQ-SF), a 7-item questionnaire that captures activity over the previous seven days [30]. It is brief, easy to administer, and reduces respondent burden when assessing total PA [30]. The IPAQ-SF was culturally adapted following IPAQ guidelines to include regionally relevant activities [31]. The total metabolic equivalent minutes per week (MET.minutes/week) and total sedentary time in minutes were calculated using the official scoring tool [31]. The participants were grouped into three activity levels on the basis of established criteria: low (not meeting moderate or high thresholds), moderate (at least 600 MET-minutes/week), and high (either 1500 MET-minutes/week of vigorous activity or 3000 MET-minutes/week from any activity) [31].

Dyspnoea severity was graded using the mMRC dyspnoea severity scale, a short patient-reported outcome in which patients choose a descriptor that best reflects their condition [32]. Scores range from grade 0 (least symptomatic) to grade 4 (most symptomatic) and can be easily administered in clinical settings [32].

### Statistical analysis

Statistical analysis was carried out using Jamovi software version 2.3.21. Continuous variables were presented as mean (SD) or median (Q1, Q3) and categorical variables as frequencies and percentages. Data normality was assessed using the Shapiro-Wilk test. Correlation analysis was carried out using the Pearson correlation coefficient test or Spearman’s rank correlation coefficient test. We classified the strength of the correlations on the basis of Rowntree’s criteria: an r < 0.2 very weak, 0.2–0.4 weak, 0.4–0.7 moderate, 0.7–0.9 strong, and >0.9 very strong [33]. Independent t tests or Mann-Whitney U tests were used to compare continuous variables between sexes. Fisher’s exact test was used to compare categorical data between sexes. Predictors of the 6MWD were identified through multivariate linear regression. Variables significant in univariate analyses (p < 0.05) were included, with the highest correlation coefficient retained in cases of multicollinearity. Multivariate regression modelling was restricted to functional exercise capacity (6MWD). Physical activity measured using IPAQ-SF was analysed using correlational methods because it represented a self-reported behavioural construct with a skewed distribution, influenced by sociocultural and contextual factors not comprehensively captured in the present dataset.

The required sample size was 55, assuming α = 0.05 and β = 0.2. Given that the strength of the correlations may vary among the variables examined in this study, the least expected correlation coefficient was conservatively set at 0.37. This was based on the lowest correlation coefficient previously reported between the incremental shuttle walk distance and PA (measured using pedometer) [6]. This calculation was primarily intended to support the correlational objectives of the study. Secondary analysis, including sex-based comparisons and multivariate regression models, should therefore be interpreted as exploratory and may have been underpowered to detect smaller effects.

## Results

Fifty-five individuals aged between 20 and 65 years with stable bronchiectasis were included. The predominant cause of bronchiectasis was post-tuberculosis. Most participants received bronchodilator medications, many also received mucolytic agents, and one participant received long-term oxygen therapy. Table 1 and S1 Table (Supporting Information) present the participants’ demographics, and clinical characteristics.

**Table 1:** Demographics, clinical and functional characteristics of the participants (n = 55).

Characteristics	Values
Age, in years (Mean ± SD)	50 ± 11
Sex (Male: Female)	21: 34
Body Mass Index, in kg/m^2^ (Mean ± SD)	21.4 ± 4.5
Occupation, n (%) Home maker Manual work Retired Desktop work Driver Shopkeeper Professionals Unemployed Other^a^	27 (49.1) 5 (9.0) 5 (9.0) 4 (7.3) 3 (5.5) 3 (5.5) 3 (5.5) 2 (3.6) 3 (5.5)
Smoking status, n (%) Non-smoker Ex – smoker Smoker Non-smoker but biomass exposure	52 (94.6) 1 (1.8) 1 (1.8) 1 (1.8)
**Disease related variables**	
Modified Medical Research Council scale for dyspnoea severity, n (%) Grade 1 Grade 2 Grade 3	39 (70.9) 12 (21.8) 4 (7.3)
Severity of bronchiectasis, n (%) Severe Moderate Mild	21 (38.2) 14 (25.4) 20 (36.4)
Radiological severity (BSI radiology score), n (%) Score 0 Score 1 Not available	20 (36.4) 33 (60.0) 2 (3.6)
Sputum microbiology, n (%) No significant growth Pseudomonas Staphylococcus Others Not available	31 (56.4) 10 (18.9) 2 (3.6) 6 (10.9) 6 (10.9)
Number of exacerbations in last year, n (%) 0 exacerbation 1 exacerbation > 1 exacerbation	20 (36.4) 22 (40.0) 13 (23.6)
Comorbidities, n (%) No comorbidities Respiratory comorbidities Cardiometabolic comorbidities Others Multiple categories	11 (20.0) 8 (14.5) 3 (5.5) 4 (7.3) 29 (52.7)
Duration since diagnosis, in years, Median, (Q1, Q3)	2 (1,8)
**Functional and physiological outcomes**	
Spirometry measures^b^ FEV1%, Median, (Q1, Q3) FVC %, Median, (Q1, Q3) FEV1/FVC, Median, (Q1, Q3)	47 (34.5–65.0) 59 (45.5–70.0) 84 (71.5–91.0)
Resting peripheral oxygen saturation, in %, Median, (Q1, Q3)	97 (95, 98)
Bronchiectasis health questionnaire total score (Mean ± SD)	61.9 ± 10.5
Absolute 6MWD, in metres (Mean ± SD)	393.0 ± 94.4
% predicted 6MWD, (Mean ± SD)	92.8 ± 20.3
Total PA, in MET.minutes/week, Median, (Q1, Q3)	3732 (1613, 5684)

^a^includes a businessman, a peon and a student; ^b^available only for 39 participants

Abbreviations: 6MWD, six-minute walk distance; % predicted 6MWD, percentage predicted six-minute walk distance; BSI, bronchiectasis severity index; FEV1, forced expiratory volume in 1 second; FVC, forced vital capacity; MET, metabolic equivalent; PA, physical activity; Q1, first quartile; Q3, third quartile.

### Relationships between PA and demographic, clinical and functional characteristics

The relationships between PA and demographic, clinical and functional characteristics are presented in Table 2. Lower physical activity levels were weakly associated with greater symptom burden, shorter disease duration, and lower percentage predicted functional exercise capacity.

**Table 2:** Relationships between PA and various variables (n = 55).

Correlations with total MET.minutes/week	r_s_ value	p value
Age in years	0.017	0.899 [NS]
BMI in kg/m^2^	0.180	0.180 [NS]
mMRC dyspnoea severity grading	−0.344	0.010
Disease duration in years	0.275	0.042
Bronchiectasis severity index category	−0.018	0.894 [NS]
Absolute 6MWD in metres	0.093	0.500 [NS]
% predicted 6MWD	0.320	0.017
BHQ score	0.252	0.063 [NS]

Abbreviations: 6MWD, six-minute walk distance; BHQ, bronchiectasis health questionnaire; BMI, body mass index; MET, metabolic equivalents; mMRC, modified Medical Research Council, NS, not significant; r_s_, Spearman’s rank correlation coefficient

### Sex-based differences in demographic, clinical and functional characteristics

Age did not differ between male and female participants (mean difference of 1.9 years, 95% CI −4.3 to 8.1 years). Compared with male participants, female participants had a higher BMI (mean difference of 2.71 kg/m^2^, 95% CI 0.29 to 5.12 kg/m^2^). There was no significant difference in disease duration between females and males (mean difference of 0.188 years, 95% CI −2.00 to 2.00 years). There was no significant association between sex and the dyspnoea severity distribution (p = 0.278) or disease severity (p = 0.686). Female participants were more physically active (total MET.minutes/week) than males (mean difference of 1350.6 MET.minutes/week, 95% CI 42–2945 MET.minutes/week) and walked shorter 6MWD compared to male participants (mean difference of −57 m, 95% CI −107.7 to −6.4 m). However, there was no significant difference in % predicted 6MWD between sexes (mean difference of 9.3%, 95% CI −0.6 to 19.1%). There was no difference in the total BHQ score between sexes (mean difference of −0.1 units, 95% CI −6.1 to 5.8 units).

Given that physical activity represents a behavioural construct influenced by sociocultural, occupational, and contextual factors beyond physiological impairment alone, predictors of functional exercise capacity (6MWD) were examined separately as a clinically distinct objective.

### Predictors of functional exercise capacity

According to the univariate analyses, lower functional exercise capacity (6MWD) was associated with older age, greater dyspnoea severity (mMRC), a higher number of exacerbations, and greater BSI category (Table 3). In contrast, better health status (BHQ) was positively associated with higher 6MWD. BMI and disease duration were not significantly associated with 6MWD.

**Table 3:** Correlations of the 6MWD with demographic and clinical variables.

6MWD	Correlation coefficient (r/r_s_)	p value
Age in years (r_s_)	−0.348	0.009
BMI in kg/m^2^ (r)	−0.084	0.543 [NS]
mMRC (r_s_)	−0.452	0.001
Disease duration in years (r_s_)	−0.006	0.963 [NS]
Bronchiectasis severity index category (r_s_)	−0.455	< 0.001
Number of exacerbations in last year (r_s_)	−0.320	0.017
BHQ score (r)	0.323	0.016
Physical activity in MET.minutes/week (r_s_)	0.093	0.500 [NS]

Abbreviations: 6MWD, six-minute walk distance; MET, metabolic equivalent; mMRC, modified Medical Research Council; r, Pearson correlation coefficient; r_s_, Spearman’s rank correlation coefficient

There was no significant difference in 6MWD between participants with and without Pseudomonas colonization (n = 49; Mann–Whitney U = 178, p = 0.770). In contrast, functional exercise capacity differed significantly between participants radiological severity as assessed by the radiological component of the BSI (n = 53; Mann–Whitney U = 199, p = 0.017), with those demonstrating greater radiological involvement walking approximately 60 m less (95% CI 14–103 m).

For multivariate linear regression, a primary model was constructed including mMRC, age, sex, and BHQ based on clinical relevance and univariate associations. The BSI category was not included due to its composite nature and conceptual overlap with dyspnoea severity, with the potential for multicollinearity. A separate model was specified to include bronchiectasis-specific variables (exacerbation frequency and radiological severity) alongside dyspnoea severity.

The primary model accounted for 33.7% of the variance in 6MWD (adjusted R² = 0.337) and demonstrated that higher mMRC grade was the only statistically significant independent predictor of reduced 6MWD (Table 4). Inspection of model residuals confirmed approximate normality and limited multicollinearity.

**Table 4:** Multivariate linear regression analysis for factors associated with the 6MWD.

Predictor	Estimate	CI	t-value	p-value
Intercept	452.58	268.75 to 636.42	4.94	<0.001
mMRC dyspnoea severity grade	−57.39	−94.99 to −19.78	−3.06	0.004
BHQ	1.60	−0.525 to 3.72	1.51	0.137
Age	−1.89	−3.83 to 0.05	−1.96	0.056
Sex (male compared with female)	37.20	−6.90 to 81.30	1.69	0.096

Note: Female sex was used as the reference category for sex.

Abbreviations: BHQ, bronchiectasis health questionnaire; CI, confidence interval; mMRC, modified Medical Research Council.

To explore potential sex-specific differences, an interaction term (sex × mMRC) was added to the model. The interaction was not statistically significant (p = 0.561), indicating that the relationship between dyspnoea severity and functional exercise capacity was similar in males and females.

In a model excluding mMRC, BHQ, age, and sex were identified as significant predictors, explaining 22.7% of the variance in 6MWD (adjusted R² = 0.227). This pattern indicates that dyspnoea severity (mMRC) is the dominant predictor of functional exercise capacity, attenuating the apparent contributions of age, sex, and overall health status.

In a model restricted to participants with available radiological data (n = 53), greater dyspnoea severity and radiological severity were independently associated with lower 6MWD. Each increase in mMRC grade was associated with an approximately 83 m reduction in 6MWD, while greater radiological severity was associated with an approximately 59 m reduction in 6MWD. Exacerbation frequency was not independently associated with 6MWD. The model explained 36.7% of the variance in functional exercise capacity (adjusted R² = 0.367). Detailed regression results are provided in S2 Table.

## Discussion

This study examined the relationships between PA and demographic, clinical and functional characteristics in individuals with stable bronchiectasis, assessed sex-based differences, and identified predictors of functional exercise capacity. Greater dyspnoea severity, shorter disease duration and lower percentage predicted functional exercise capacity were associated with lower PA levels. Females reported higher PA despite shorter absolute walking distances, although relative functional exercise capacity (% predicted 6MWD) did not differ significantly between sexes. Dyspnoea severity emerged as the strongest predictor of functional exercise capacity, while radiological severity was also independently associated with reduced 6MWD.

Greater dyspnoea severity was weakly associated with lower PA, consistent with dyspnoea being an unpleasant sensation that may lead individuals to avoid activities that provoke symptoms [6,34,35]. This finding aligns with previous studies reporting associations between lower PA and higher dyspnoea grades in bronchiectasis [6,11]. Unlike those studies [6,11], which used pedometers in larger cohorts, our study employed a self-report questionnaire. Although device-based assessments provide objective PA data, questionnaire-based tools such as the IPAQ-SF remain more feasible in many South Asian and resource-limited settings because they are low cost, less burdensome, and easier to integrate into routine clinical evaluation, particularly where healthcare interactions are episodic and long-term follow-up may be limited [15,36–38].

Individuals with longer disease duration reported slightly higher PA levels. This may partly reflect the skewed distribution of disease duration in our sample and behavioural adaptation following diagnosis of chronic illness [39]. Repeated healthcare interactions over time may improve symptom understanding, pacing strategies, and confidence in maintaining activity levels [34]. However, this finding should be interpreted cautiously given the self-reported nature of PA assessment and the weak correlation observed.

Regular PA, even at moderate levels, is generally associated with improvements in exercise capacity through enhanced mitochondrial function, greater muscle strength and improved cardiovascular efficiency [40]. While several previous studies have reported associations between PA and functional exercise capacity in bronchiectasis [7,11–13], we observed no relationship between PA and absolute 6MWD. Similarly, Cakmak et al.[5], found no association between PA and walking distance despite differences between the studies in the expression of functional exercise capacity, PA assessment methods, and cohort characteristics. In contrast, we observed a weak positive correlation between PA and % predicted 6MWD, similar to Jose et al.[6], who reported a moderate association between number of steps per day and % predicted ISWT. These findings suggest that relative functional performance adjusted for individual characteristics may better reflect habitual activity levels than absolute walking distance alone. Nevertheless, the weak strength of the association indicates that functional exercise capacity alone may not necessarily translate into higher real-world PA, highlighting the multidimensional determinants of PA in bronchiectasis. Together, these findings suggest that PA behaviour and functional exercise capacity, although related, may represent distinct constructs in bronchiectasis that do not necessarily share identical determinants.

Interestingly, PA was not related to BMI, differing from earlier bronchiectasis studies using accelerometers [7,12]. This discrepancy may reflect differences in cohort characteristics and the greater proportion of female homemakers in our sample, whose domestic activities may be better captured by self-report questionnaires than by hip-worn monitors. Evidence from non-bronchiectasis populations suggests that self-report questionnaires may better capture contextual domestic activities than hip-worn monitors [41,42].

In this study, females had poorer functional exercise capacity (absolute 6MWD) than males; however, no significant sex difference was observed in % predicted 6MWD after adjustment for age and sex. This suggests that the lower absolute walking distance may partly reflect expected anthropometric and physiological sex differences rather than disproportionate disease-related functional impairment [18,29]. In contrast, females reported higher PA levels, which may reflect sociocultural and occupational roles within this cohort, particularly household and caregiving activities captured by questionnaire-based assessments such as IPAQ-SF. These findings highlight the importance of interpreting self-reported PA within its sociocultural context.

Greater dyspnoea severity emerged as the strongest independent predictor of reduced 6MWD, consistent with previous findings in bronchiectasis [13,17] and other respiratory diseases [43,44]. These findings are consistent with the rationale for interventions such as pulmonary rehabilitation [45], airway clearance techniques [46], or bronchodilator therapy [47], aimed at symptom reduction. When dyspnoea was excluded from the model, younger age, male sex, and better health status were associated with greater walking distance, suggesting that dyspnoea may attenuate the apparent contribution of these variables to functional exercise capacity. In the model restricted to participants with available radiological data, radiological severity was also independently associated with lower 6MWD. Greater structural lung involvement may contribute to impaired ventilation, mucus retention, airflow limitation, and increased symptom burden, thereby limiting exercise performance. Similar associations between HRCT abnormalities, radiological extent of disease, and reduced functional exercise capacity have previously been reported in bronchiectasis [17,18].

### Strength and limitations

This study assessed individuals with bronchiectasis from a low- and middle-income country with sociocultural contexts that differ from those in high-income settings. Such factors may influence overall PA levels and symptom perception. The findings should therefore be generalized with caution. As participants were recruited as part of a pulmonary rehabilitation trial, the cohort may represent a relatively more motivated and clinically stable subgroup than the broader bronchiectasis population. Although the sample size was calculated for the correlational objectives of the study, it may have been underpowered for some secondary analyses, particularly sex-based subgroup comparisons and multivariate regression modelling. Additionally, PA was measured using a self-report questionnaire, which is prone to recall bias and may incorrectly estimate PA [5,48]. The IPAQ-SF is also known to overestimate moderate-intensity physical activity, particularly when domestic and caregiving activities are interpreted as moderate intensity exercise. This consideration may be especially relevant in South Asian contexts, where household responsibilities performed predominantly by women may substantially contribute to self-reported PA levels. Nevertheless, questionnaire-based assessments remain the most feasible approach in this regional context. Additionally, disease severity classification used a modified or relative BSI approach in participants with incomplete conventional BSI variables. Although similar pragmatic adaptations have previously been reported in real-world bronchiectasis cohorts, [24] the use of relative categorisation rather than complete conventional BSI scoring may have introduced some degree of severity misclassification and should be interpreted with caution.

Our findings support previous evidence that improvements in functional exercise capacity do not automatically translate into increased PA levels. Pulmonary rehabilitation programs should therefore extend beyond improving exercise performance to address behavioural and contextual factors influencing daily activity participation. Sex-specific considerations may also be important, as males and females may differ in baseline capacity, sociocultural roles, symptom perception, and activity behaviour.

## Conclusion

In individuals with bronchiectasis, lower PA levels were associated with greater dyspnoea severity, shorter disease duration, and lower percentage predicted functional exercise capacity. Females reported higher self-reported PA levels despite shorter absolute walking distances than males, although relative functional exercise capacity did not differ significantly between sexes. Dyspnoea severity emerged as the strongest predictor of functional exercise capacity, while radiological severity was also independently associated with reduced 6MWD. Further studies should examine longitudinal relationships among disease progression, symptoms, and functional exercise capacity and explore the underlying reasons for sex differences in PA and symptom perception. Future research should further explore the behavioural and sociocultural factors influencing PA participation and rehabilitation engagement in bronchiectasis.

## Supporting information

S1 Table Additional patient characteristics. Abbreviations: COPD, chronic obstructive pulmonary disease; PA, physical activity; Q1, first quartile; Q3, third quartile.
(DOCX)

S2 Table Multivariable linear regression model for six-minute walk distance among participants with available radiological data (n = 53). Abbreviations: 6MWD, six-minute walk distance; mMRC, modified Medical Research Council dyspnoea scale; CI, confidence interval; SE, standard error. *Radiological severity was categorized based on the presence of involvement of ≥3 lobes and/or cystic bronchiectasis on computed tomography.
(DOCX)

## References

[1] Aliberti S, Goeminne PC, O’Donnell AE, Aksamit TR, Al-Jahdali H, Barker AF, et al. Criteria and definitions for the radiological and clinical diagnosis of bronchiectasis in adults for use in clinical trials: international consensus recommendations. Lancet Respir Med. 2022;10(3):298–306. doi: 10.1016/S2213-2600(21)00277-0 34570994

[2] Shteinberg M, Flume PA, Chalmers JD. Is bronchiectasis really a disease?. Eur Respir Rev. 2020;29(155):190051. doi: 10.1183/16000617.0051-2019 31996354 PMC9488738

[3] Ozalp O, Inal-Ince D, Calik E, Vardar-Yagli N, Saglam M, Savci S, et al. Extrapulmonary features of bronchiectasis: muscle function, exercise capacity, fatigue, and health status. Multidiscip Respir Med. 2012;7(1):3. doi: 10.1186/2049-6958-7-3 22958327 PMC3415114

[4] Dudgeon EK, Crichton M, Chalmers JD. “The missing ingredient”: the patient perspective of health related quality of life in bronchiectasis: a qualitative study. BMC Pulm Med. 2018;18(1):81. doi: 10.1186/s12890-018-0631-7 29788953 PMC5964675

[5] Cakmak A, Inal-Ince D, Sonbahar-Ulu H, Bozdemir-Ozel C, Ozalp O, Calik-Kutukcu E, et al. Physical activity of patients with bronchiectasis compared with healthy counterparts: a cross-sectional study. Heart Lung. 2020;49(1):99–104. doi: 10.1016/j.hrtlng.2019.09.004 31530430

[6] José A, Ramos TM, de Castro RAS, de Oliveira CS, de Camargo AA, Athanazio RA, et al. Reduced physical activity with bronchiectasis. Respir Care. 2018;63(12):1498–505. doi: 10.4187/respcare.05771 30254043

[7] Bradley JM, Wilson JJ, Hayes K, Kent L, McDonough S, Tully MA, et al. Sedentary behaviour and physical activity in bronchiectasis: a cross-sectional study. BMC Pulm Med. 2015;15:61. doi: 10.1186/s12890-015-0046-7 25967368 PMC4456779

[8] Alcaraz-Serrano V, Arbillaga-Etxarri A, Oscanoa P, Fernández-Barat L, Bueno L, Amaro R, et al. Exacerbations and Changes in Physical Activity and Sedentary Behaviour in Patients with Bronchiectasis after 1 Year. J Clin Med. 2021;10(6):1190. doi: 10.3390/jcm10061190 33809173 PMC7998500

[9] Alcaraz-Serrano V, Gimeno-Santos E, Scioscia G, Gabarrús A, Navarro A, Herrero-Cortina B, et al. Association between physical activity and risk of hospitalisation in bronchiectasis. Eur Respir J. 2020;55(6):1902138. doi: 10.1183/13993003.02138-2019 32184321

[10] Pitta F, Burtin C. The physical activity coach in pulmonary rehabilitation. Textbook of pulmonary rehabilitation. Springer International Publishing; 2017. 195–204. doi: 10.1007/978-3-319-65888-9_15

[11] de Camargo AA, Boldorini JC, Holland AE, de Castro RAS, Lanza F de C, Athanazio RA, et al. Determinants of peripheral muscle strength and activity in daily life in people with bronchiectasis. Phys Ther. 2018;98(3):153–61. doi: 10.1093/ptj/pzx123 29237078

[12] Cordova-Rivera L, Gibson PG, Gardiner PA, McDonald VM. Physical activity associates with disease characteristics of severe asthma, bronchiectasis and COPD. Respirology. 2019;24(4):352–60. doi: 10.1111/resp.13428 30384396

[13] de Camargo AA, Amaral TS, Rached SZ, Athanazio RA, Lanza FC, Sampaio LM, et al. Incremental shuttle walking test: a reproducible and valid test to evaluate exercise tolerance in adults with noncystic fibrosis bronchiectasis. Arch Phys Med Rehabil. 2014;95(5):892–9. doi: 10.1016/j.apmr.2013.11.019 24361325

[14] Cordova-Rivera L, Gibson PG, Gardiner PA, Hiles SA, McDonald VM. Extrapulmonary associations of health status in severe asthma and bronchiectasis: comorbidities and functional outcomes. Respir Med. 2019;154:93–101. doi: 10.1016/j.rmed.2019.06.010 31229944

[15] Sylvia LG, Bernstein EE, Hubbard JL, Keating L, Anderson EJ. Practical guide to measuring physical activity. J Acad Nutr Diet. 2014;114(2):199–208. doi: 10.1016/j.jand.2013.09.018 24290836 PMC3915355

[16] Yildiz S, Inal-Ince D, Calik-Kutukcu E, Vardar-Yagli N, Saglam M, Arikan H, et al. Clinical determinants of incremental shuttle walk test in adults with bronchiectasis. Lung. 2018;196(3):343–9. doi: 10.1007/s00408-018-0094-x 29435737

[17] Guan W-J, Gao Y-H, Xu G, Lin Z-Y, Tang Y, Li H-M, et al. Six-minute walk test in Chinese adults with clinically stable bronchiectasis: association with clinical indices and determinants. Curr Med Res Opin. 2015;31(4):843–52. doi: 10.1185/03007995.2015.1013625 25708564

[18] Lee AL, Button BM, Ellis S, Stirling R, Wilson JW, Holland AE, et al. Clinical determinants of the 6-Minute Walk Test in bronchiectasis. Respir Med. 2009;103(5):780–5. doi: 10.1016/j.rmed.2008.11.005 19070473

[19] Jacques PS, Gazzana MB, Palombini DV, Barreto SSM, Dalcin P de TR. Six-minute walk distance is not related to quality of life in patients with non-cystic fibrosis bronchiectasis. J Bras Pneumol. 2012;38(3):346–55. doi: 10.1590/s1806-37132012000300010 22782605

[20] Sami R, Zohal M, Mohammadi N. Clinical determinants of the Six-Minute Walk Test (6MWT) in stable non-cystic fibrosis bronchiectasis patients. Tanaffos. 2020;19(4):385–91. 33959177 PMC8088139

[21] Buran MM, Savci S, Tanriverdi A, Kahraman BO, Gunduz D, Sevinc C. Clinical determinants of the modified incremental step test in adults with non-cystic fibrosis bronchiectasis. J Bras Pneumol. 2024;50(1):e20230230. doi: 10.36416/1806-3756/e20230230 38422338 PMC11095920

[22] Choi H, Xu J-F, Chotirmall SH, Chalmers JD, Morgan LC, Dhar R. Bronchiectasis in Asia: a review of current status and challenges. Eur Respir Rev. 2024;33(173):240096. doi: 10.1183/16000617.0096-2024 39322263 PMC11423131

[23] Chalmers JD, Goeminne P, Aliberti S, McDonnell MJ, Lonni S, Davidson J, et al. The bronchiectasis severity index. An international derivation and validation study. Am J Respir Crit Care Med. 2014;189(5):576–85. doi: 10.1164/rccm.201309-1575OC 24328736 PMC3977711

[24] Howarth T, Gibbs C, Abeyaratne A, Heraganahally SS. Applicability and validity of the “Bronchiectasis Severity Index” (BSI) and “FACED” score in adult aboriginal Australians. Int J Chron Obstruct Pulmon Dis. 2024;19:2611–28. doi: 10.2147/COPD.S482848 39650744 PMC11625430

[25] Spinou A, Siegert RJ, Guan W-J, Patel AS, Gosker HR, Lee KK, et al. The development and validation of the Bronchiectasis Health Questionnaire. Eur Respir J. 2017;49(5):1601532. doi: 10.1183/13993003.01532-2016 28495688

[26] Committee on Proficiency Standards for Clinical Pulmonary Function Laboratories ATS. ATS statement: guidelines for the six-minute walk test. Am J Respir Crit Care Med. 2002;166(1):111–7. doi: 10.1164/ajrccm.166.1.at1102 12091180

[27] Holland AE, Spruit MA, Troosters T, Puhan MA, Pepin V, Saey D, et al. An official European Respiratory Society/American Thoracic Society technical standard: field walking tests in chronic respiratory disease. Eur Respir J. 2014;44(6):1428–46. doi: 10.1183/09031936.00150314 25359355

[28] Williams N. The Borg Rating of Perceived Exertion (RPE) scale. Occup Med-Oxford. 2017;67(5):404–5. doi: 10.1093/occmed/kqx063

[29] Agarwal B, Shah M, Sawant B, Bagwe H, Murkudkar P, Mullerpatan R. Predictive equation for six-minute walk test in Indian children, adolescents, and adults. Lung India. 2023;40(2):143–8. doi: 10.4103/lungindia.lungindia_680_21 37006098 PMC10174647

[30] Lee PH, Macfarlane DJ, Lam TH, Stewart SM. Validity of the International Physical Activity Questionnaire Short Form (IPAQ-SF): a systematic review. Int J Behav Nutr Phys Act. 2011;8:115. doi: 10.1186/1479-5868-8-115 22018588 PMC3214824

[31] IPAQ Website administrator. IPAQ - Adapt 2022. Accessed 2024 July 25. https://sites.google.com/view/ipaq/adapt

[32] Mahler DA, Wells CK. Evaluation of clinical methods for rating dyspnea. Chest. 1988;93(3):580–6. doi: 10.1378/chest.93.3.580 3342669

[33] Overholser BR, Sowinski KM. Biostatistics primer: part 2. Nutr Clin Pract. 2008;23(1):76–84. doi: 10.1177/011542650802300176 18203967 10.1177/011542650802300176

[34] Royle H, Kelly C. “The likes of me running and walking? No chance”: exploring the perceptions of adult patients with bronchiectasis towards exercise. Chronic Illn. 2023;19(1):157–71. doi: 10.1177/17423953221108223 35695195 PMC9843538

[35] Lavery K, O’Neill B, Elborn JS, Reilly J, Bradley JM. Self-management in bronchiectasis: the patients’ perspective. Eur Respir J. 2007;29(3):541–7. doi: 10.1183/09031936.00057306 17079260

[36] Maher C, Szeto K, Arnold J. The use of accelerometer-based wearable activity monitors in clinical settings: current practice, barriers, enablers, and future opportunities. BMC Health Serv Res. 2021;21(1):1064. doi: 10.1186/s12913-021-07096-7 34625076 PMC8501528

[37] Strath SJ, Kaminsky LA, Ainsworth BE, Ekelund U, Freedson PS, Gary RA, et al. Guide to the assessment of physical activity: clinical and research applications: a scientific statement from the American Heart Association. Circulation. 2013;128(20):2259–79. doi: 10.1161/01.cir.0000435708.67487.da 24126387

[38] Sharma J, Pavlova M, Groot W. Challenges and opportunities for universal health coverage in south asia: a scoping review. Asia Pac J Public Health. 2025;37(1):7–16. doi: 10.1177/10105395241296653 39545483 PMC11800725

[39] Bonino S, Calandri E, Cattelino E. Living with a chronic illness as a challenge to psychological development: the role of personal identity, sense of coherence and perceived self-efficacy. Soc Sci Human Open. 2025;11:101620. doi: 10.1016/j.ssaho.2025.101620

[40] Spagnolo A, Klug S, Schenkl C, Schwarzer M. Links between exercise capacity, exercise training, and metabolism. Comp Phys. 2023;13(4):5115–55. doi: 10.1002/j.2040-4603.2023.tb00283.x

[41] Shiroma EJ, Schepps MA, Harezlak J, Chen KY, Matthews CE, Koster A, et al. Daily physical activity patterns from hip- and wrist-worn accelerometers. Physiol Meas. 2016;37(10):1852–61. doi: 10.1088/0967-3334/37/10/1852 27654140 PMC5360542

[42] Ahmad MH, Salleh R, Mohamad Nor NS, Baharuddin A, Rodzlan Hasani WS, Omar A, et al. Comparison between self-reported physical activity (IPAQ-SF) and pedometer among overweight and obese women in the MyBFF@home study. BMC Womens Health. 2018;18(Suppl 1):100. doi: 10.1186/s12905-018-0599-8 30066635 PMC6069802

[43] Spruit MA, Watkins ML, Edwards LD, Vestbo J, Calverley PMA, Pinto-Plata V, et al. Determinants of poor 6-min walking distance in patients with COPD: the ECLIPSE cohort. Respir Med. 2010;104(6):849–57. doi: 10.1016/j.rmed.2009.12.007 20471236

[44] Foglio K, Carone M, Pagani M, Bianchi L, Jones PW, Ambrosino N. Physiological and symptom determinants of exercise performance in patients with chronic airway obstruction. Respir Med. 2000;94(3):256–63. doi: 10.1053/rmed.1999.0734 10783937

[45] Lee AL, Gordon CS, Osadnik CR. Exercise training for bronchiectasis. Cochrane Database Syst Rev. 2021;4(4):CD013110. doi: 10.1002/14651858.CD013110.pub2 33822364 PMC8093919

[46] Üzmezoğlu B, Altıay G, Özdemir L, Tuna H, Süt N. The efficacy of flutter® and active cycle of breathing techniques in patients with bronchiectasis: a prospective, randomized, comparative study. Turk Thorac J. 2018;19(3):103–9. doi: 10.5152/TurkThoracJ.2018.17050 30083399 PMC6077004

[47] Cazzola M, Martínez-García MÁ, Matera MG. Bronchodilators in bronchiectasis: there is light but it is still too dim. Eur Respir J. 2022;59(6):2103127. doi: 10.1183/13993003.03127-2021 35680152

[48] O’Neill B, McDonough SM, Wilson JJ, Bradbury I, Hayes K, Kirk A, et al. Comparing accelerometer, pedometer and a questionnaire for measuring physical activity in bronchiectasis: a validity and feasibility study?. Respir Res. 2017;18(1):16. doi: 10.1186/s12931-016-0497-2 28088206 PMC5237513

