# Supplementary material for: Physical activity, sex differences, and functional exercise capacity in bronchiectasis: Insights from a South Asian cohort
Source: PLoS One. 2026 Sep 2;21(9):e0356560. doi: 10.1371/journal.pone.0356560 (PMC13537580; doi:10.1371/journal.pone.0356560)
Supplement: Supplementary file 1 [file pone.0356560.s001.docx]

**Title: Physical activity, sex differences, and functional exercise capacity in bronchiectasis: insights from a South Asian cohort**

**S1 Table. Additional patient characteristics (n = 55)**

| **Cause of bronchiectasis, n (%)** | |
| --- | --- |
| Post tuberculosis  Post-infectious  Bronchial Asthma  Allergic bronchopulmonary aspergillosis  Unknown  Chronic obstructive pulmonary disease  Asthma – COPD overlap syndrome  Mixed etiology | 24 (43.6)  12 (21.8)  9 (16.4)  3 (5.5)  3 (5.5)  2 (3.6)  1 (1.8)  1 (1.8) |
| **Physical activity levels** | |
| PA level, High, n (%) | 32 (58.2%) |
| PA level, Moderate, n (%) | 21 (38.2%) |
| PA level, Low, n (%) | 2 (3.6%) |
| Total sedentary time, in minutes/day, Median (Q1, Q3) | 180 (120, 308) |
| **Treatment related variables** | |
| On long-term oxygen therapy, n (%) | 1 (1.8) |
| On Long-acting beta agonist, n (%) | 38 (69.1) |
| On short-acting beta agonist, n (%) | 4 (7.3) |
| On combination of long- and short-acting beta-agonist, n (%) | 1 (1.8) |
| Mucolytic agents, n (%) | 25 (45.5) |

Abbreviations: COPD, chronic obstructive pulmonary disease; PA, physical activity; Q1, first quartile; Q3, third quartile.
