# Supplementary material for: Physical activity, sex differences, and functional exercise capacity in bronchiectasis: Insights from a South Asian cohort
Source: PLoS One. 2026 Sep 2;21(9):e0356560. doi: 10.1371/journal.pone.0356560 (PMC13537580; doi:10.1371/journal.pone.0356560)
Supplement: Supplementary file 2 [file pone.0356560.s002.docx]

**Title: Physical activity, sex differences, and functional exercise capacity in bronchiectasis: insights from a South Asian cohort**

**S2 Table. Multivariable linear regression model for six-minute walk distance among participants with available radiological data (n = 53)**

| **Predictor** | **Estimate** | **CI** | **t-value** | **p-value** |
| --- | --- | --- | --- | --- |
| Intercept | 550.04 | 491.7 to 608.4 | 18.950 | <0.001 |
| mMRC | -82.98 | -117.6 to -48.3 | -4.813 | <0.001 |
| Exacerbation frequency | -2.97 | -19.2 to 13.3 | -0.367 | 0.715 |
| Radiological severity* | -58.94 | -106.2 to -11.7 | -2.508 | 0.015 |

Abbreviations: 6MWD, six-minute walk distance; mMRC, modified Medical Research Council dyspnoea scale; CI, confidence interval; SE, standard error.

*Radiological severity was categorized based on the presence of involvement of ≥3 lobes and/or cystic bronchiectasis on computed tomography.
